# Factors and outcomes associated with acute kidney injury in brain tumor resection patients: insights from a large US database (2010–2019)

**DOI:** 10.1080/0886022X.2025.2587502

**Published:** 2025-11-24

**Authors:** Binbin Tian, Mingdi Chen, Junfen Cheng, Jian Wang, Boqi Geng, Junde Mo, Guorong Zhong, Zhugui Chen

**Affiliations:** ^a^ Department of Critical Care Medicine, Zhanjiang Central Hospital, Guangdong Medical University, Zhanjiang, Guangdong, China; ^b^ Department of Critical Care Medicine, The Second Affiliated Hospital of Guangdong Medical University, Zhanjiang, Guangdong, China; ^c^ Department of Respiration, The Second Affiliated Hospital of Guangdong Medical University, Zhanjiang, Guangdong, China; ^d^ Division of Orthopaedic Surgery, Department of Orthopaedics, Nanfang Hospital, Southern Medical University, Guangzhou, Guangdong, China; ^e^ Department of Anesthesiology, Zhanjiang Central Hospital, Guangdong Medical University, Zhanjiang, Guangdong, China

**Keywords:** Brain tumor resection, acute kidney injury, risk factors, Nationwide Inpatient Sample

## Abstract

Acute kidney injury (AKI) is a major perioperative complication following brain tumor resection, yet multi-center studies on this topic remain scarce. This study aimed to determine the incidence, risk factors, and clinical outcomes associated with AKI in patients undergoing brain tumor resection, utilizing a nationally representative dataset. We analyzed brain tumor resection admissions from the United States’ National Inpatient Sample database (2010–2019), identifying hospitalizations with and without AKI using International Classification of Diseases, Ninth Revision, Clinical Modification and International Classification of Diseases, Tenth Revision, Clinical Modification codes. Multivariable logistic regression analyses were performed to evaluate the associations between patient/hospital characteristics, comorbidities, complications and AKI. Among more than 40,000 brain tumor resection admissions, AKI occurred in 3.1% of hospitalizations, with prevalence rising from 1.8% in 2010 to 4.4% in 2019. AKI-associated admissions had higher costs (median $171,904 vs. $99,821, *p* < .001), longer hospital stays (median 11 vs. 5 days), increased mortality (8.3% vs. 1.2%, *p* < .001), higher dialysis requirement (1.1% vs. 0.01%, *p* < .001), and mechanical ventilation (12.1% vs. 3.0%, *p* < .001). Associated risk factors for AKI included: age ≥65 years, Black/Hispanic race, congestive heart failure, diabetes, fluid and electrolyte disorders, other neurological disorders, obesity, and chronic kidney disease excluding end-stage renal disease. Medical complications associated with increased AKI risk included septicemia, deep vein thrombosis, urinary tract infection, pneumonia, and cerebral edema. Female sex and elective admission were protective factors. Prompt identification of these risk factors is crucial for optimizing perioperative management and improving clinical outcomes.

## Background

Primary brain and central nervous system (CNS) tumors are the second most common cancer type among adolescents and young adults, and they rank eighth in the older population [1]. For most primary brain neoplasms, the initial therapeutic approach focuses on maximal surgical removal, which serves both diagnostic purposes and improves survival outcomes [2]. Surgery-induced alterations in neuroendocrine function, impaired tissue perfusion, inflammatory processes, and preexisting health conditions collectively affect kidney function and homeostasis [3,4].

Major surgery frequently triggers acute kidney injury (AKI), leading to adverse physiological responses, including fluid imbalance, renin-angiotensin-aldosterone pathway activation, inflammatory mediator release, and metabolic disruption [4–6]. These perturbations can result in serious complications such as cardiac events, septicemia, and coagulation disorders [6,7]. Moreover, patients who develop AKI face increased risks of extended hospitalization, ICU admission, ventilator dependence, chronic kidney disease progression, and higher healthcare costs [4,8]. Given these detrimental outcomes, preventing postoperative AKI and identifying its risk factors represent essential clinical imperatives. In brain tumor surgeries, established risk factors include preoperative mannitol use, hypoalbuminemia, and postoperative hyperchloremia [7–9].

Nevertheless, prior studies on perioperative AKI in brain tumor surgery have been limited to small, single-center cohorts. This gap was addressed in our study through a nationwide retrospective analysis to identify risk factors for perioperative AKI in patients undergoing brain tumor surgery.

### Data source

Our study utilized records from the Nationwide Inpatient Sample (NIS), part of the Healthcare Cost and Utilization Project overseen by the Agency for Healthcare Research and Quality. The NIS is the largest inpatient database in the United States, encompassing all payment sources and sampling roughly 20% of annual hospitalizations from over 1,000 medical facilities, representing approximately 7 million hospitalizations per year[10]. This database includes detailed clinical and administrative data, covering patient characteristics, hospital features, economic indicators, and diagnostic/procedure codes. Since we used de-identified, publicly accessible data, ethical review was not required, in accordance with Helsinki Declaration guidelines.

### Data collection

**Figure 1.**
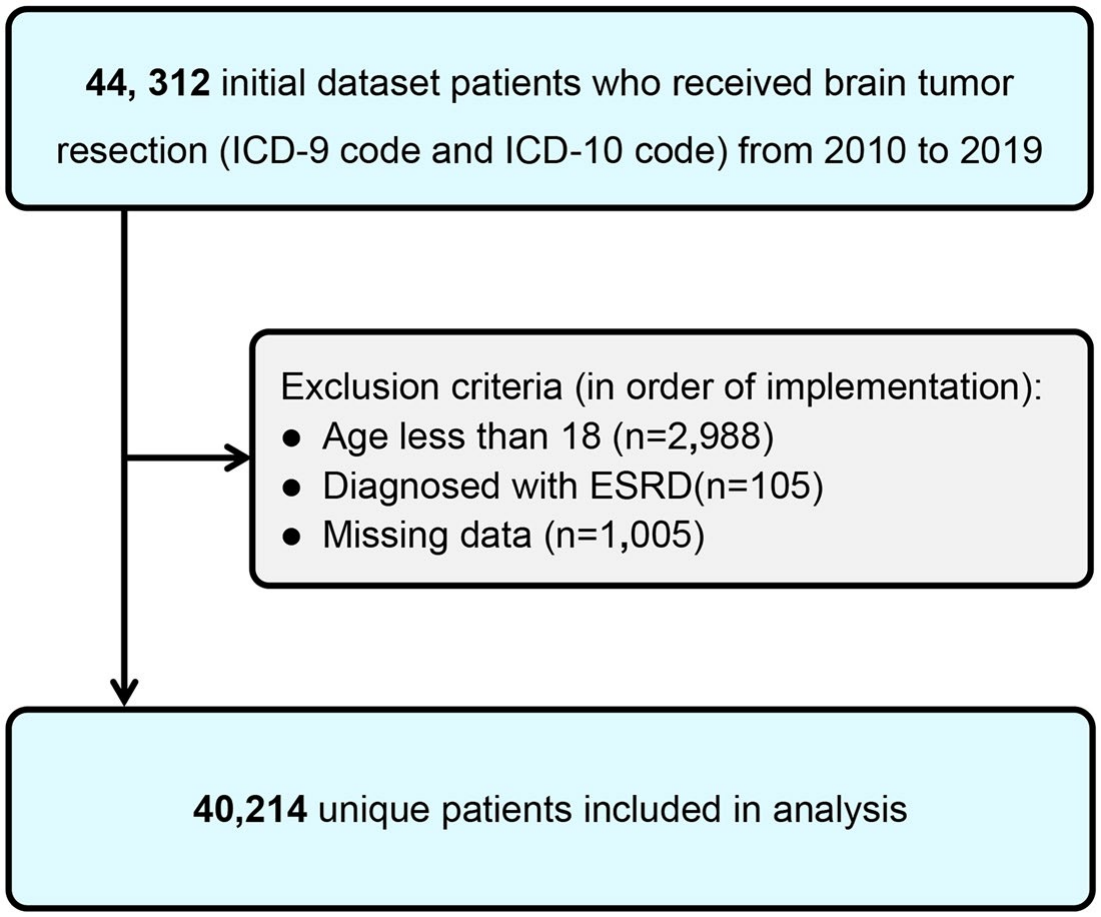
Inclusion/exclusion process of brain tumor resection.

We analyzed NIS records from 2010 to 2019, focusing on brain tumor craniotomy admissions. Relevant hospitalizations were identified using International Classification of Diseases, Ninth Revision, Clinical Modification (ICD-9-CM) and International Classification of Diseases, Tenth Revision, Clinical Modification (ICD-10-CM) codes for meningiomas, gliomas, and metastatic brain lesions (Supplementary Table 1). Surgical interventions were tracked using procedure codes for operations on the brain, cerebral hemispheres, cerebellum, meninges, dura mater, cerebral ventricles, pons, and medulla oblongata (Supplementary Table 1). AKI was identified using the following diagnostic codes: ICD-9-CM codes (584/584.5/584.6/584.7/584.8/584.9) and ICD-10-CM codes (N17, N17.0, N17.1, N17.2, N17.8, N17.9) [10]. We excluded patients under 18 years of age, those with end-stage renal disease (ESRD), and those with incomplete documentation (Figure 1).

The cohort was divided into two groups: those with perioperative AKI and those without. We compared patient demographics, hospital characteristics, comorbidities, complications, and clinical outcomes between these groups. Key outcomes included length of hospital stay, treatment costs, mortality rates, and the need for ventilator support or dialysis. Comorbidities and complications were documented using standardized diagnostic categories (Table 1).

**Table 1.** Variables used in binary logistic regression analysis.

Variables categories	Specific variables
Patient demographics	Age (≤64 years and ≥65 years), sex (male and female), race (White, Black, Hispanic, Asian or Pacific Islander, Native American, and Other)
Hospital characteristics	Type of admission (non-elective, elective), bed size of hospital (small, medium, large), teaching status of hospital (nonteaching, teaching), location of hospital (rural, urban), type of insurance (Medicare, Medicaid, private insurance, self-pay, no charge, other), location of the hospital (northeast, Midwest or north central, south, west)
Comorbidities	AIDS, aids, alcohol abuse, deficiency anemia, rheumatoid arthritis/collagen vascular diseases, chronic blood loss anemia, congestive heart failure, chronic pulmonary disease, coagulopathy, depression, diabetes, drug abuse, hypertension, hypothyroidism, liver disease, lymphoma, fluid and electrolyte disorders, metastatic cancer, other neurological disorders, obesity, paralysis, peripheral vascular disorders, psychoses, pulmonary circulation disorders, chronic kidney disease excluding ESRD, peptic ulcer disease, valvular disease, weight loss
Complications	Medical complications (septicemia, acute myocardial infarction, deep vein thrombosis, urinary tract infection, cardiac arrest, urinary retention, pneumonia, respiratory failure, heart failure, pulmonary embolism, thrombocytopenia, cerebral edema); Surgical complications (wound infection, hemorrhage, seroma, hematoma)

*Note:* AIDS: acquired immunodeficiency syndrome; ESRD: end-stage renal disease.

### Statistical analysis

Statistical analysis was performed using SPSS version 25. Continuous variables were compared using independent t-tests, and categorical variables were analyzed with chi-square tests. Multivariable logistic regression was conducted to identify independent predictors of perioperative AKI, incorporating patient demographics, institutional features, and documented medical conditions (Table 1). The analysis produced odds ratios with 95% confidence intervals, and *p* < .001 was considered statistically significant [10].

## Result

### Temporal trends and overall incidence

**Figure 2.**
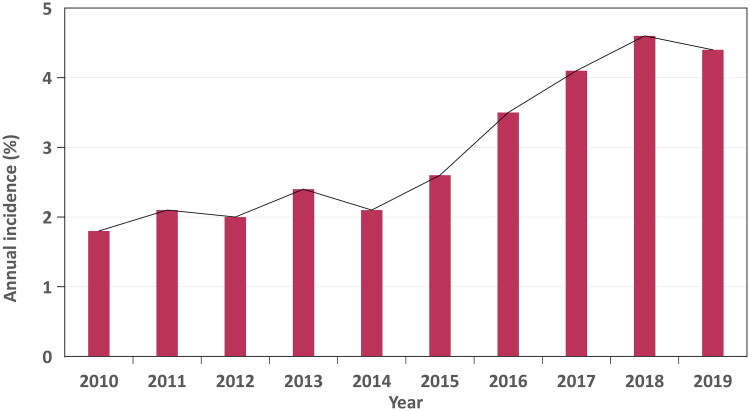
Annual incidence of AKI following brain tumor resection.

A retrospective analysis from 2010 to 2019 included 40,214 patients undergoing brain tumor resection, of whom 1,247 (3.1%) developed perioperative AKI. The incidence increased significantly over time, from 1.8% at the start of the study to 4.4% by its conclusion (Figure 2).

### Patient demographics and hospital characteristics

Patients who developed AKI were significantly older, with a mean age difference of 8 years (67 vs. 59 years, *p* < .001). AKI was more common in the 65–74 age group, with an 11.6 percentage point increase (34.6% vs. 23.0%, *p* < .001). AKI patients were more likely to be male than non-AKI patients (64.6% vs. 48.2%, *p* < .001). Patients with AKI often had a higher number of comorbidities (≥3 comorbidities: 85.4% vs. 44.1%, *p* < .001) (Table 2).

**Table 2.** Patient characteristics and outcomes in brain tumor resection (2010–2019).

Characteristics	AKI	No AKI	*p*
Total (*n* = count)	1,247	38,967	
Total incidence	3.1		
Age (median, years)	67 (59, 75)	59 (47, 68)	<.001
Age group			
18–44	85 (6.8%)	8,119 (20.8%)	<.001
45–64	394 (31.6%)	17,689 (45.4%)	
65–74	432 (34.6%)	8,946 (23.0%)	
≥75	336 (26.9%)	4,213 (26.9%)	
Sex			
Male	806 (64.6%)	18,783 (48.2%)	<.001
Female	441 (35.4%)	20,184 (51.8%)	
Race			
White	780 (62.6%)	27,940 (71.7%)	<.001
Black	207 (16.6%)	3,225 (8.3%)	
Hispanic	114 (9.1%)	3,069 (7.9%)	
Asian or Pacific Islander	29 (2.3%)	1,054 (2.7%)	
Native American	4 (0.3%)	123 (0.3%)	
Other	113 (9.1%)	3,556 (9.1%)	
Number of comorbidity			
0	17 (1.4%)	6,414 (16.5%)	<.001
1	59 (4.7%)	7,957 (20.4%)	
2	106 (8.5%)	7,414 (19.0%)	
≥3	1,065 (85.4%)	17,179 (44.1%)	
Type of insurance			
Medicare	727 (58.3%)	13,632 (35.0%)	<.001
Medicaid	111 (8.9%)	4,492 (11.5%)	
Private insurance	321 (25.7%)	18,190 (46.7%)	
Self-pay	38 (3.0%)	1,295 (3.3%)	
No charge	4 (0.3%)	125 (0.3%)	
Other	46 (3.7%)	1,233 (3.2%)	
Bed size of hospital			
Small	88 (7.1%)	2,635 (6.8%)	<.001
Medium	232 (18.6%)	6,735 (17.3%)	
Large	927 (74.3%)	29,578 (75.9%)	
Region of hospital			
Northeast	226 (18.1%)	7,767 (19.9%)	
Midwest or North Central	271 (21.7%)	8,323 (21.4%)	
South	509 (40.8%)	14,734 (37.8%)	
West	241 (19.3%)	8,143 (20.9%)	
Elective admission	227 (22.2%)	21,362 (54.8%)	<.001
Type of hospital (teaching)	1,065 (85.4%)	33,822 (86.8%)	<.001
Location of hospital (urban)	1,230 (98.6%)	38,454 (98.7%)	<.001
Died	103 (8.3%)	1.2 (1.2%)	<.001
LOS (median, d)	11 (7, 18)	5(3, 8)	<.001
TOTCHG (median, $)	171,904 (109,554- 279,402)	99,821 (65,099– 156,567)	<.001
Dialysis	14 (1.1%)	4 (0.01%)	<.001
Mechanical ventilation	151 (12.1%)	1,151 (3.0%)	<.001

*Note:* AKI: acute kidney injury; LOS: length of stay; TOTCHG: total charge.

A facility-based evaluation revealed higher AKI rates in hospitals across the Midwest/North Central (21.7% vs. 21.4%, *p* < .001), and South (40.8% vs. 37.8%, *p* < .001). Perioperative AKI following brain tumor resection tended to occur in medium size hospital (18.6% vs. 17.3%, *p <* .001). Lower incidence rates were observed among White patients (62.6% vs. 71.7%, *p* < .001), teaching hospitals (85.4% vs. 86.8%, *p* < .001), and large hospitals (74.3% vs. 75.9%, *p* < .001). Medicare was the predominant insurer for AKI hospitalizations (58.3% vs. 35.0%, *p* < .001), while private insurance (25.7% vs. 46.7%) and Medicaid (8.9% vs. 11.5%) were less common (*p* < .001). Elective admissions were associated with a lower incidence of AKI (22.2% vs. 54.8%, *p* < .001) (Table 2).

### Health outcomes following AKI

Perioperative AKI was associated with worse clinical outcomes, including longer hospitalization (median 11 vs. 5 days, *p* < .001), higher treatment costs ($171,904 vs. $99,821, *p* < .001), and increased mortality (8.3% vs. 1.2%, *p* < .001). These patients also had a higher need for mechanical ventilation (12.1% vs. 3.0%, *p* < .001) and dialysis support (1.1% vs. 0.01%, *p* < .001) (Table 2).

### Independent risk factors for AKI identified through multivariate analysis

**Figure 3.**
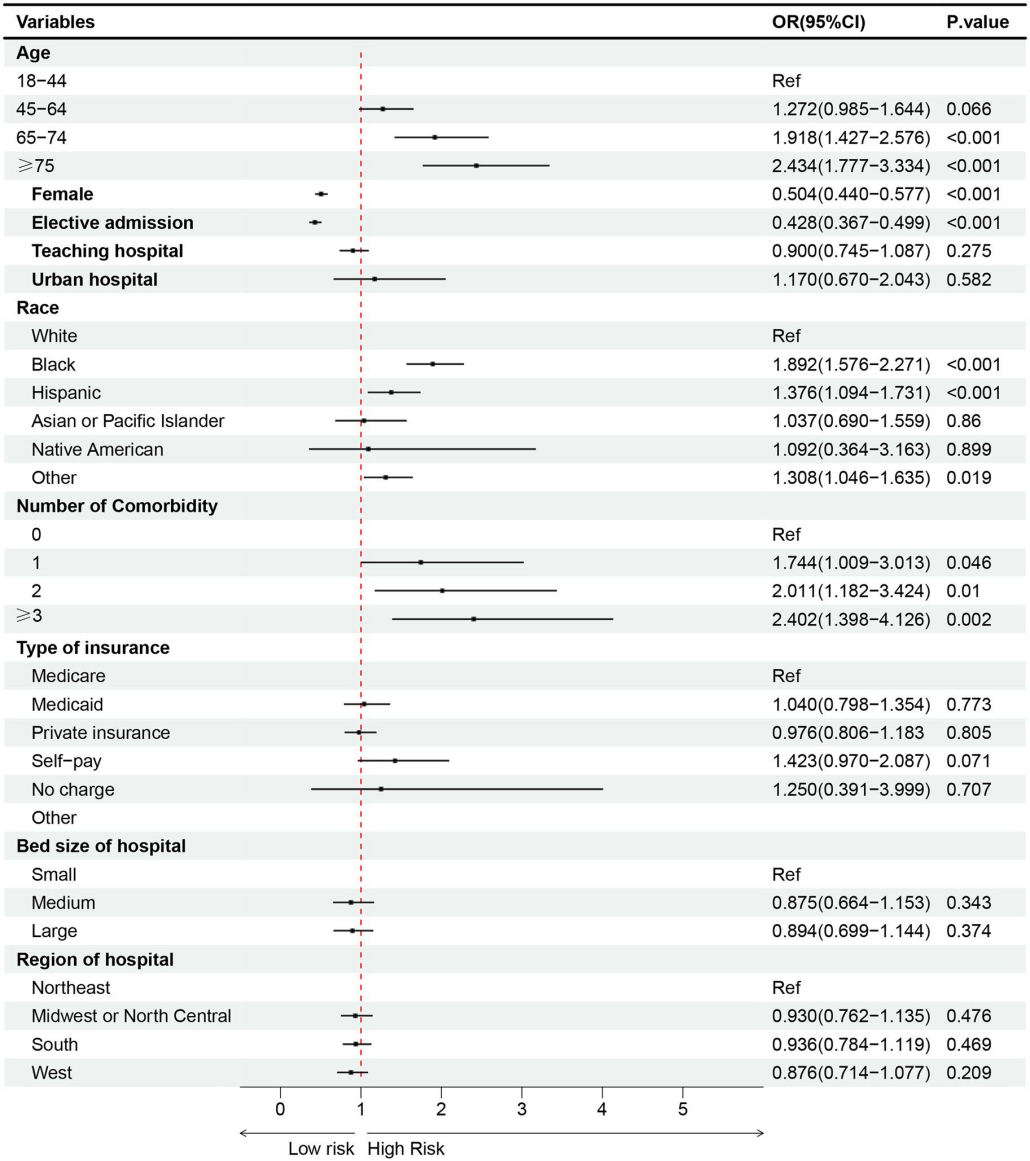
Risk factors associated with to AKI in brain tumor resection.

**Figure 4.**
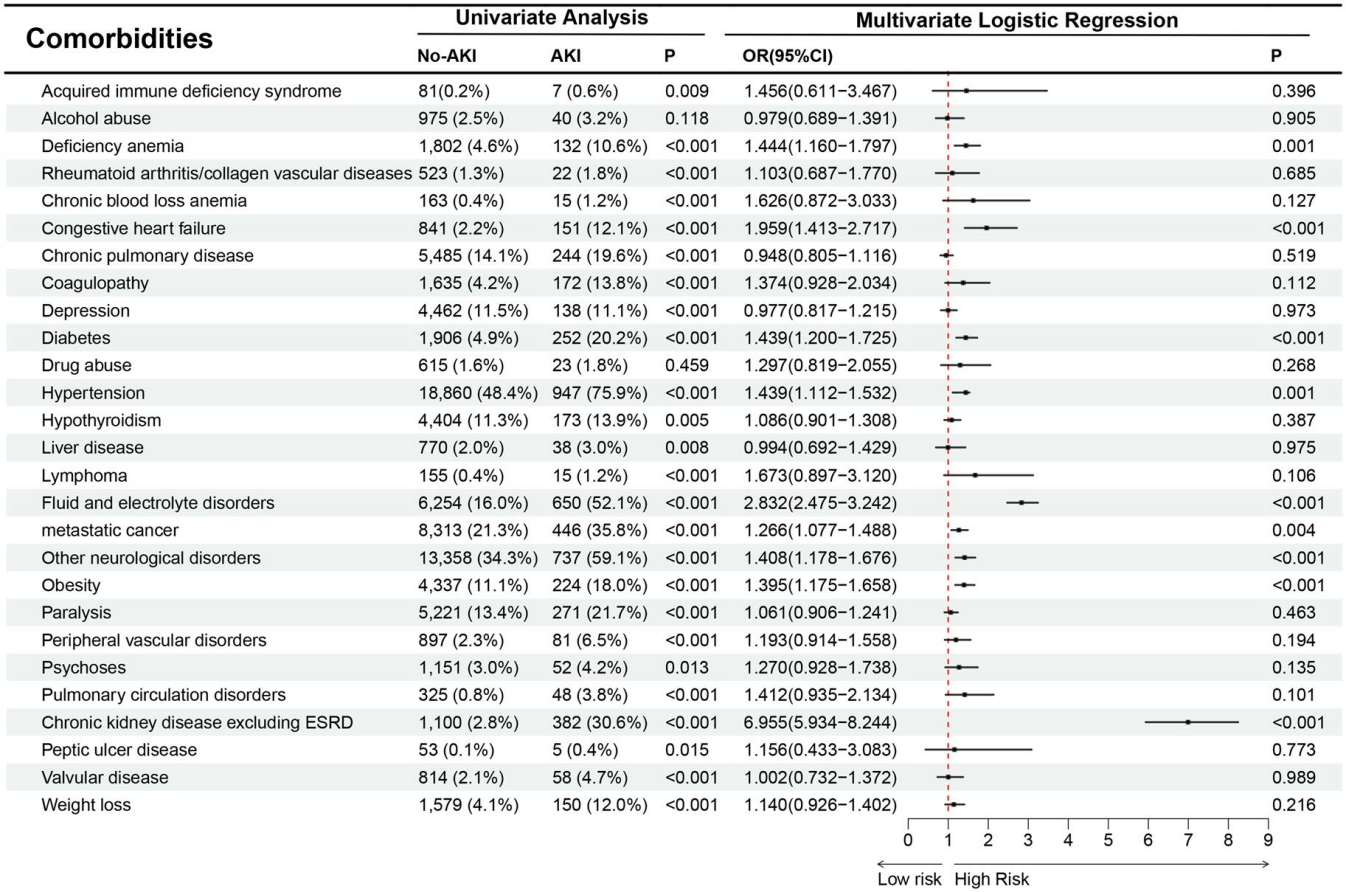
Relationship between AKI and comorbidities.

Multivariable logistic analysis identified significant risk factors associated with AKI, including advanced age (≥65 years), Black/Hispanic race, and comorbidities (congestive heart failure, diabetes, fluid and electrolyte disorders, neurological disorders, obesity, and chronic kidney disease (CKD) excluding ESRD (Figures 3 and 4). Female sex and elective admission were found to be protective factors (Figure 3).

### Complications associated with AKI following brain tumor resection

**Figure 5.**
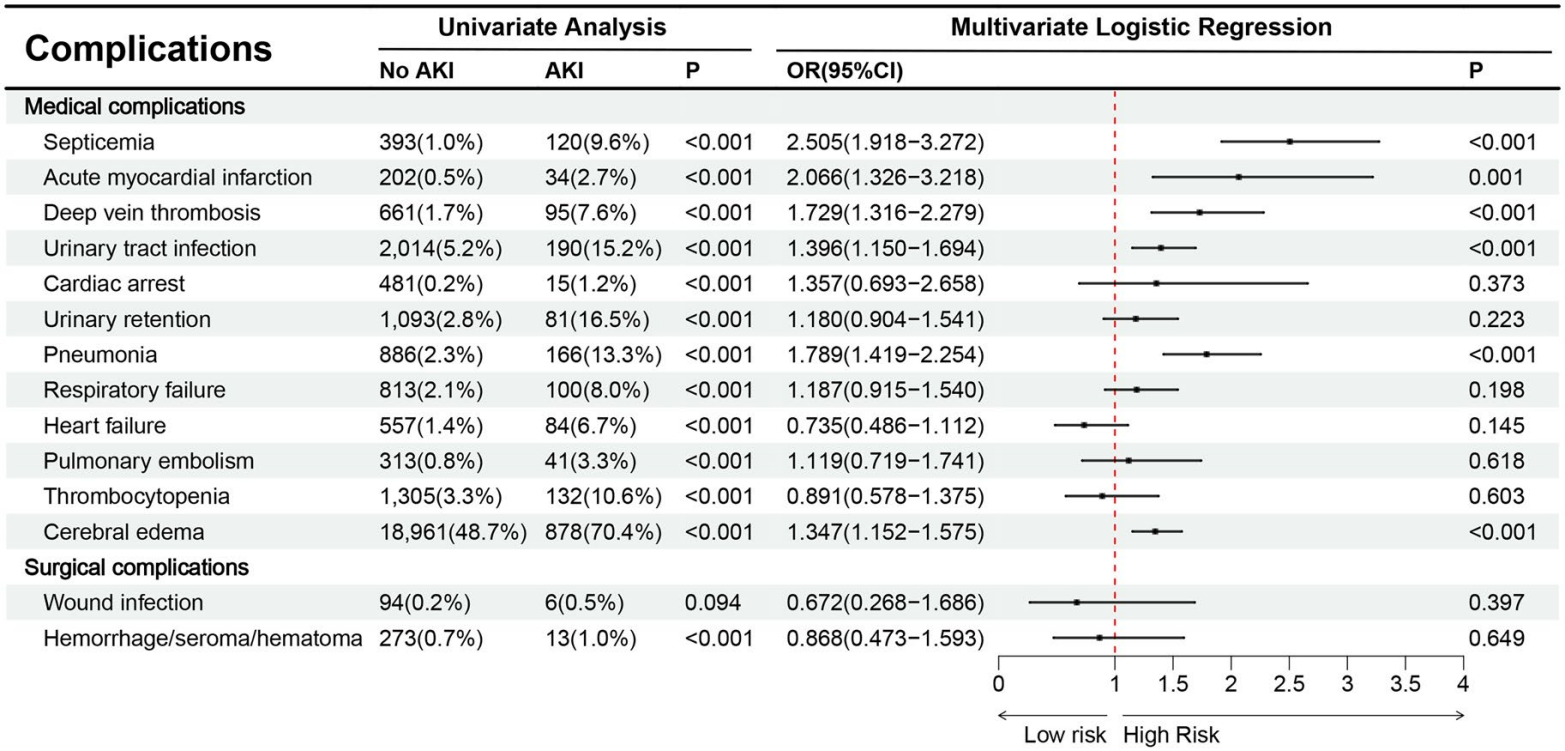
Relationship between AKI and complications.

Multivariable analysis highlighted several complications significantly associated with perioperative AKI, including septicemia, deep vein thrombosis, urinary tract infection, pneumonia, and cerebral edema (Figure 5).

## Discussion

### Key findings

Our analysis of more than 40,000 brain tumor resection-related admissions from a nationally representative US sample represents, to our knowledge, the largest cohort study to date on AKI incidence and risk factors in this population. The findings reveal significant associations between AKI following brain tumor resection and increased in-hospital mortality, as well as a higher demand for invasive interventions. Notably, we observed variability in patient characteristics between AKI and non-AKI cases and provide insight into key trends in brain tumor resection admissions by AKI status over time (Figure S1). This study is the first comprehensive analysis of perioperative AKI among brain tumor resection admissions utilizing the NIS database after the transition to ICD-10 coding, providing contemporary national insights into these hospitalizations.

### Incidence

In the nationwide analysis of the 2010–2019 US NIS, AKI was diagnosed in 3.1% of all brain tumor resection hospitalizations, a rate slightly diverging from previously reported ranges (1.16%, 1.8%, and 5.4%) [7–9]. These discrepancies can be related to differences in surgical type, urgency, study populations, AKI diagnostic criteria, and the distinctions between clinical and administrative diagnoses [3,4,11,12]. The observed increasing trend in AKI-related incidence following brain tumor resection during the study period is in line with previous research [10,13]. First, the heightened awareness of AKI as a post-neurosurgical complication has led to improved identification and coding of previously unrecognized mild cases [14]. The sensitivity of administrative codes, as shown by Grams et al. which improved from 10% to 24%, supports this finding [15]. Second, the recurrent nature of AKI may contribute to higher readmission rates, thereby elevating the observed incidence [14,16]. Lastly, the increasing prevalence of comorbidities, such as hypertension, obesity, and diabetes—recognized AKI risk factors—may help explain these trends [15]. These findings underline the need for a deeper understanding of AKI mechanisms in brain tumor resection and for enhanced recognition and management strategies [14].

### Adverse outcome

AKI is associated with severe adverse outcomes, including elevated in-hospital mortality, prolonged hospital stays, and increased healthcare costs. AKI also triggers complications like acute respiratory and cardiac failure, often necessitating invasive interventions such as mechanical ventilation and dialysis [13,14,17]. Given the high mortality and significant healthcare resource utilization associated with AKI, early intervention is crucial upon diagnosis. Management strategies should include immediate resuscitation, nephrology consultation, and optimized dialysis protocols in intensive care settings [11,14]. This requires coordinated multidisciplinary collaboration among nephrologists, pharmacists, and rehabilitation teams to implement comprehensive care plans [18].

### Risk factors

This large-scale investigation not only highlights the poor outcomes of AKI following brain tumor resection but also identifies key risk factors. Advanced age was found to be a significant predictor of AKI, in agreement with previous studies [19]. Aging increases AKI susceptibility through both structural and functional renal decline, including nephron loss and reduced renal blood flow [19]. Elderly patients typically exhibit impaired hemodynamic adaptability and reduced renal reserve, conditions exacerbated by comorbidities such as hypertension and diabetes, which increase their vulnerability to nephrotoxins and dehydration [20,21]. Huang et al. showed that age-related glomerulosclerosis and reduced physiologic tolerance interact synergistically to elevate perioperative AKI risk [20].

The analysis identified Black and Hispanic races as independent risk factors for post-brain tumor resection AKI. Lunyera et al. highlighted remain increased AKI odds among Black patients, even after adjusting for relevant clinical variables[22]. Multicenter studies by Bjornstad et al. suggested that these disparities may stem from unequal healthcare access, a disproportionate chronic disease burden, and potential genetic or unmeasured social determinants, such as neighborhood deprivation [23]. Further research is necessary to explore these underlying factors and to develop targeted interventions that address racial disparities in AKI outcomes [22].

Diabetes significantly increases AKI risk through mechanisms such as hyperglycemia-induced inflammation, oxidative stress, and renal hypoperfusion [20,21,24]. Hapca et al. found that diabetic patients without CKD are approximately five times more likely to develop AKI compared to those without diabetes [20]. Effective perioperative AKI risk mitigation requires comprehensive preoperative blood glucose assessment, rigorous glucose and renal function monitoring, and maintenance of glucose levels between 110 and 149 mg/dL, a range linked to reduced AKI incidence and stable mortality outcomes [16].

Our analysis shows that CKD patients have a 6.995-fold increased risk of AKI. CKD consistently appears as a major risk factor across medical and surgical interventions [25,26]. Patients with both CKD and hypertension have a particularly heightened risk for AKI, due to reduced renal reserve and complications associated with diuretic therapy or renin-angiotensin-aldosterone system inhibitors. As Bradshaw et al. have noted, effective AKI prevention in this population requires meticulous management of these pharmacologic and physiological factors [25]. Additionally, our study confirms congestive heart failure as an AKI risk factor, potentially mediated by reduced renal perfusion pressure, resulting in pre-renal injury [26].

In our cohort, obesity emerged as a significant risk factor for AKI. This finding is consistent with Huang et al.’s meta-analysis, which showed that elevated body mass index (BMI ≥25 kg/m^2^) increases postoperative AKI risk in colorectal cancer surgery patients [20]. The impact of obesity on AKI risk is particularly significant in perioperative settings, driven by multiple mechanisms: metabolic syndrome, altered renal hemodynamics, and obesity-related glomerulopathy, characterized by glomerulomegaly and alterations in glomerular filtration rate [27].

Fluid and electrolyte disorders were additional critical risk factors for AKI in our study. Oh et al. showed that hyperchloremia (serum chloride ≥110 mmol/L) independently predicts AKI development, potentially linked to chloride-rich fluid administration, which induces hyperchloremic acidosis and subsequent renal tubular injury [8]. Chen et al. identified various electrolyte disturbances, including hypernatremia and hypokalemia etc., as correlates of AKI [28]. Kumar et al. demonstrated that each 1 mEq/L increase in serum sodium corresponds to a 5.4% increase in AKI risk among subarachnoid hemorrhage patients [29]. Furthermore, Wang et al. reported that fluid overload exceeding 10% of total volume increases AKI incidence, whereas goal-directed fluid therapy may reduce perioperative AKI risk [30]. These findings emphasize the critical importance of precise hemodynamic management in reducing postoperative AKI risk.

### Protective factor

Sex differences and admission type play a significant role in influencing the risk of post-brain tumor resection AKI. Female appear to have a protective effect against AKI, supported by experimental data showing that testosterone induces increased oxidative stress, inflammation, and structural renal changes in males. Conversely, estrogen in females enhances antioxidant defenses and maintains endothelial stability [20,31]. The observed male predisposition to AKI may also be partially explained by diagnostic factors, as higher baseline serum creatinine levels in males could lead to an overestimation of AKI incidence when compared to females [14]. Additionally, elective admissions are associated with a reduced risk of AKI, likely associated with optimized preoperative management and a generally healthier baseline status. On the other hand, urgent or emergent surgeries, often linked to advanced disease and hemodynamic instability, substantially increase the likelihood of AKI [10,32].

### Clinical and research implications

This study analyzed a large cohort of brain tumor resection patients over a decade, allowing for a comprehensive assessment of AKI epidemiology, risk factors, and clinical outcomes. The investigation utilized recent NIS data to examine clinical differences between patients with and without AKI, contributing to the existing body of literature. The inclusion of hospitals from both rural and urban settings enhances the generalizability of the findings to the broader US population undergoing brain tumor resection. This research identifies potential perioperative risk mitigation strategies, including structured patient education, avoidance of nephrotoxic agents, optimization of fluid management, targeted comorbidity management, and the implementation of standardized AKI care pathways. However, prospective validation studies are necessary to assess the combined effectiveness of these interventions in clinical practice.

### Limitation

While our study possesses several strengths, it also has inherent limitations due to the nature of administrative datasets. Discharge coding captures only the first 15 diagnoses, which could introduce potential bias. Furthermore, the transition from ICD-9 to ICD-10 may result in diagnostic classification variability, despite efforts to cross-map [15,21]. The administrative coding system counts AKI diagnoses per admission rather than per patient, which may lead to an overestimation of incidence due to duplicate entries, while also underestimating the true burden of AKI due to underdiagnosis and the reliance on less sensitive ICD codes compared to laboratory-based criteria. Temporal changes in coding sensitivity and evolving clinical awareness may skew trends in incidence, although the increasing rates of AKI in brain tumor resections suggest a genuine shift in epidemiology. Additionally, the NIS lacks detailed information on surgery type, operation duration, anesthesia-related data, intraoperative transfusion, and granular laboratory results, particularly serum creatinine levels, which hinders accurate assessment of baseline kidney function, AKI staging, and renal recovery. The dataset’s lack of data on chronic kidney disease heterogeneity and fluid/electrolyte parameters further limits pathophysiological insights. While these limitations prevent causal inferences and detailed risk stratification, the strengths of the dataset in capturing large-scale prognostic patterns emphasize the need for prospective studies that integrate multimodal clinical data.

## Conclusion

This study underscores the significant clinical and economic burden of AKI in hospitalized patients undergoing brain tumor resection. It demonstrates a high incidence of AKI and its association with increased mortality, healthcare utilization, and modifiable risk factors. These disparities highlight the critical need for early risk stratification, evidence-based perioperative interventions, and prompt management to mitigate AKI progression, ultimately improving renal and overall postoperative outcomes for this vulnerable population.

## Supplementary Material

Supplementary Table 1.docx

Figure S1.tif

## Data Availability

This study is based on data provided by Nationwide Inpatient Sample (NIS) database, part of the Healthcare Cost and Utilization Project, Agency for Healthcare Research and Quality. The NIS database is a large publicly available full-payer inpatient care database in the United States, and the direct web link to the database is https://www.ahrq.gov/data/hcup/index.html. Therefore, individual or grouped data cannot be shared by the authors.
