# Supplementary material for: Factors and outcomes associated with acute kidney injury in brain tumor resection patients: insights from a large US database (2010–2019)
Source: Ren Fail. 2025 Nov 24;47(1):2587502. doi: 10.1080/0886022X.2025.2587502 (PMC12646087; doi:10.1080/0886022X.2025.2587502)
Supplement: Supplementary Table 1.docx [file IRNF_A_2587502_SM5724.docx]

**Supplementary Table 1**: International Classification of Diseases (9th Revision and 10th Revision) Clinical Modification (ICD-9-CM and ICD-10-CM) Codes That Were Used

| Diagnosis/Procedure | ICD-9 CM Code | ICD-10 CM Code |
| --- | --- | --- |
| Brain tumor | 2396,2250, v1085,1910-1919 | D320, D330, D331, D332, C700, C709, C71, C7931, C7932 |
| Surgical procedure | 016, 0151, 0152 0153, 0159, 0131 0132, 0139 | 00500ZZ, 00B00ZZ, 00C00ZZ, 00510ZZ, 00B10ZZ, 00C10ZZ, 00D10ZZ, 00520ZZ, 00B20ZZ, 00C20ZZ, 00D20ZZ, 00560ZZ, 00B60ZZ, 00C60ZZ, 00570ZZ, 00B70ZZ, 00C70ZZ, 00T70ZZ, 005B0ZZ, 00BB0ZZ, 00CB0ZZ, 005C0ZZ, 00BC0ZZ, 00CC0ZZ, 005D0ZZ, 00BD0ZZ, 00CD0ZZ |

ICD-9-CM, International Classification of Diseases (9th Revision) Clinical Modification.

ICD-10-CM, International Classification of Diseases (10th Revision) Clinical Modification.
