# Supplementary figures and images for: Factors and outcomes associated with acute kidney injury in brain tumor resection patients: insights from a large US database (2010–2019)
Source: Ren Fail. 2025 Nov 24;47(1):2587502. doi: 10.1080/0886022X.2025.2587502 (PMC12646087; doi:10.1080/0886022X.2025.2587502)

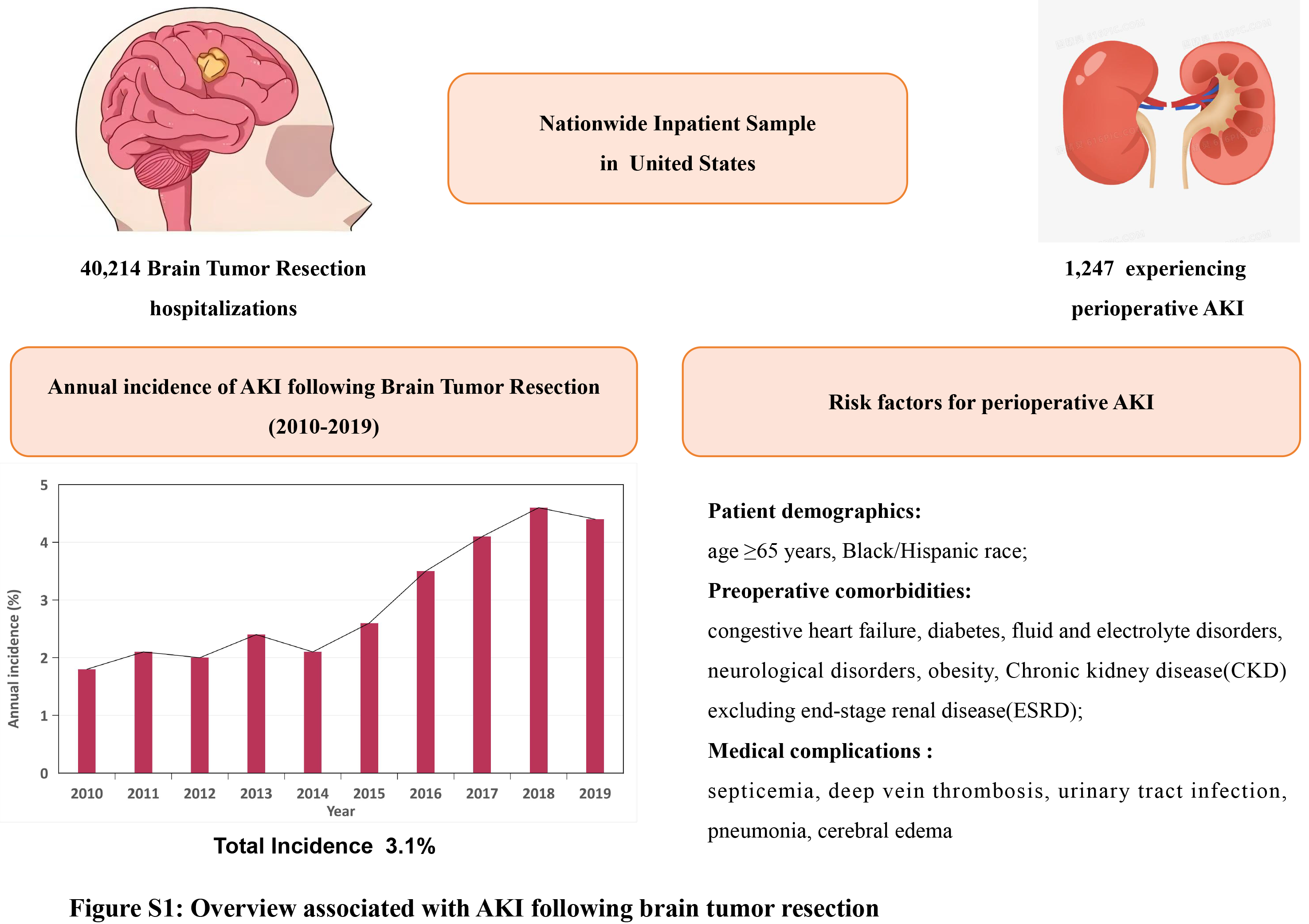

Supplement: Figure S1.tif [file IRNF_A_2587502_SM5723.tif]
